# How the Italian Twitter Conversation on Vaccines Changed During the First Phase of the Pandemic: A Mixed-Method Analysis

**DOI:** 10.3389/fpubh.2022.824465

**Published:** 2022-05-18

**Authors:** Francesco Gesualdo, Lorenza Parisi, Ileana Croci, Francesca Comunello, Andrea Parente, Luisa Russo, Ilaria Campagna, Barbara Lanfranchi, Maria Cristina Rota, Antonietta Filia, Alberto Eugenio Tozzi, Caterina Rizzo

**Affiliations:** ^1^Multifactorial and Complex Diseases Research Area, Bambino Gesù Children's Hospital, IRCCS, Rome, Italy; ^2^Department of Human Sciences, Link Campus University, Rome, Italy; ^3^Department of Communication and Social Research, Sapienza University of Rome, Rome, Italy; ^4^Department of Infectious Diseases, Istituto Superiore di Sanità, Rome, Italy; ^5^Clinical Pathways and Epidemiology Unit, Bambino Gesù Children's Hospital, IRCCS, Rome, Italy

**Keywords:** vaccines, COVID-19, vaccine hesitancy, communication, social media, Twitter

## Abstract

In the context of the European Joint Action on Vaccination, we analyzed, through quantitative and qualitative methods, a random sample of vaccine-related tweets published in Italy between November 2019 and June 2020, with the aim of understanding how the Twitter conversation on vaccines changed during the first phase of the pandemic, compared to the pre-pandemic months. Tweets were analyzed by a multidisciplinary team in terms of kind of vaccine, vaccine stance, tone of voice, population target, mentioned source of information. Multiple correspondence analysis was used to identify variables associated with vaccine stance. We analyzed 2,473 tweets. 58.2% mentioned the COVID-19 vaccine. Most had a discouraging stance (38.1%), followed by promotional (32.5%), neutral (22%) and ambiguous (2.5%). The discouraging stance was the most represented before the pandemic (69.6%). In February and March 2020, discouraging tweets decreased intensely and promotional and neutral tweets dominated the conversation. Between April and June 2020, promotional tweets remained more represented (36.5%), followed by discouraging (30%) and neutral (24.3%). The tweets' tone of voice was mainly polemical/complaining, both for promotional and for discouraging tweets. The multiple correspondence analysis identified a definite profile for discouraging and neutral tweets, compared to promotional and ambiguous tweets. In conclusion, the emergence of SARS-CoV-2 caused a deep change in the vaccination discourse on Twitter in Italy, with an increase of promotional and ambiguous tweets. Systematic monitoring of Twitter and other social media, ideally combined with traditional surveys, would enable us to better understand Italian vaccine hesitancy and plan tailored, data-based communication strategies.

## Introduction

Since 2015, the volume of articles published in the scientific literature on social media and vaccines has seen an exponential growth (1), encompassing investigations on the use of social media as a source of information on vaccines (2), research based on social media monitoring projects (3, 4) and descriptions of interventions for vaccine promotion delivered through social media (5).

The COVID-19 pandemic has intensified the involvement of social media users in the discourse on health and vaccines. The role of social media in spreading health information, both from reliable and from questionable sources, has increased during the pandemic (6), and the overabundance of information circulating on the web—defined by the World Health Organization (WHO) as the “infodemic” (7)—has made it difficult for users to find trustworthy sources of information. Moreover, social media are a means to share stories, opinions and emotions that may have an impact on health behaviors, including the intention to vaccinate (8).

In the present article, we report an in-depth analysis of a large corpus of tweets on vaccines published in Italy immediately before and during the first months of the COVID-19 pandemic. The tweet corpus was collected for a social media monitoring platform recently created by the Italian National Institute of Health, in collaboration with the Bambino Gesù Children's Hospital IRCCS (Rome, Italy), in the context of the European Joint Action on Vaccination (EU-JAV) (9).

To place the study within the historical and cultural context of Italy, in 2017 the Italian government, concerned by decreasing vaccine uptake rates, introduced compulsory vaccination for ten routine childhood vaccinations (10, 11). A positive impact of the law on vaccine uptake (12) has been reported.

The analyzed tweets were posted between November 12, 2019 (start of the data retrieval for the EU-JAV social media monitoring platform) and June 22, 2020. COVID-19 started occupying the front pages of Italian newspapers at the end of January 2020, when the WHO declared COVID-19 a “global risk” (January 28, 2020), followed by the report of a Chinese couple who tested positive in Rome (January 30, 2020). The first native Italian COVID-19 cases were reported on February 21, 2020, a national lockdown was established on March 8, and restrictions were temporarily lifted in the first half of May 2020.

In this intense period, the pandemic had an impact on risk perception and on vaccine confidence in general. On the one hand, the sense of vulnerability and fear increased people's engagement in preventive behaviors (13) and may have improved vaccine confidence in the population (14). A recent survey conducted in elderly people in Southern Italy found an association between vaccine acceptance and use of social media or mass media as a source of information on vaccines (15). On the other hand, conspiracy theories on the origin of the pandemic and on the COVID-19 vaccine might have had a negative effect on propensity to vaccination (14). The level of trust toward institutions (16), which is a crucial determinant of vaccine confidence, has also changed during the pandemic, and these changes have been context-specific (17).

Several studies explored the characteristics of the vaccine discourse on social media during the pandemic (18–21), though only few studies (22) investigated the impact of the pandemic on the vaccine-related discourse, comparing its characteristics before and during COVID-19. Our objective was to understand how the Italian conversation about vaccines on Twitter changed during the first phase of the pandemic, in terms of vaccine stance, involved actors and dominant narratives, compared to the months immediately preceding the pandemic. To this aim, we conducted a mixed-method analysis on tweets collected before and during the first months of the pandemic.

## Methods

This is a cross sectional study analyzing the vaccination discourse on Twitter in Italy between November 2019 and June 2020.

In the context of the EU-JAV, the Italian National Health Institute, in collaboration with the Bambino Gesù Children's Hospital, Rome, Italy, developed a platform aimed at monitoring vaccination discourse on social media and other web sources. Twitter data for the EU-JAV platform have been collected, using Python 3.0, through the Twitter application programmer's interfaces (API) service (standard v1.1), following the Twitter API Terms of Use, starting from November, 2019, on the basis of keyword filters that were validated following a structured framework. Through these filters, we selected vaccine-related tweets in Italian, French and Spanish. The Italian filter included one of the following keywords: vaccino OR vaccini OR vaccinata OR vaccinato OR vaccinate OR vaccinati OR vaccinazione OR vaccinazioni OR novax. The analysis was conducted on Italian tweets only.

From this database, with the purpose of acquiring an in-depth understanding of the characteristics and the variation of the vaccination discourse in Italy during the first phase of the pandemic, compared to the immediate pre-pandemic months, we selected a random sample of 3,187 tweets in Italian, posted between 15 November 2019 and 22 June 2020 (a total of 33 weeks).

In order to analyze the entire vaccine-related conversation, we decided: (a) to include in the final dataset not only original tweets, but also retweets and replies, (b) to include unverified users, and (c) not to use any software to identify users likely to be bots, given that also tweets by these users contribute to the overall vaccine discourse. For each tweet downloaded through the API, the following metadata were available: text, publishing date and hour, tweet URL. Tweets were jointly analyzed by a multidisciplinary group of researchers with expertise in health, vaccines and social sciences, namely internet studies. This was done through qualitative text analysis (23), using both deductive and inductive approaches in constructing the coding book. More specifically, tweets were classified according to selected categories identified through these approaches. The deductive categories that emerged from the published literature were vaccine stance, kind of vaccine and population target of the vaccine, while categories generated inductively were: tone of voice, source of information and kind of user. Information on the kind of user was obtained through the profile's bio.

An explanation of stance categories is reported in Table 1, and is adapted from a classification elaborated by the Vaccine Confidence Project for tweets on vaccination during pregnancy (24).

**Table 1:** Vaccine stance classification.

**Promotional** Communicate public health benefits or safety of vaccinations. Encourage vaccination. Describe risks of not vaccinating. Refute claims that vaccines are dangerous.
**Ambiguous** Contain indecision, uncertainty on the risks or benefits of vaccination. Contain disapproving and approving information.
**Neutral** Contain no elements of uncertainty, promotional or negative content regarding vaccines (may express doubts about political decisions regarding vaccination programs, but they refer to vaccines in a neutral way). Include factual recommendations about vaccines. Often statements.
**Discouraging** Contain negative attitudes/arguments against vaccines. Contain questions re. effectiveness/safety or possibility of adverse reactions that may or may not be proven. Discourage the use of recommended vaccines.
**Other** Vaccine-related tweets in which the stance cannot be understood (e.g., tweets including sarcasm or using an ambiguous language).

The tone of voice (or “tone”) category is used in media content analysis to describe the way a message is addressed, thus influencing audience reception, e.g., it is used to describe the media coverage of an event. Together with the stance, the tone of voice is very useful to contextualize the intention of the author and the latent content. Whereas, the tone of voice generally uses three labels (positive, neutral, and negative) (25) we used a richer categorization to better describe the framing of the message (see below).

In summary, each tweet was categorized according to:

- Vaccine stance: promotional, ambiguous, discouraging, critical against the anti-vax community, neutral, other
- Tone of voice: ironic, polemical/complaining, worried, supportive/empathic, purposeful, enthusiastic, neutral, other
- Kind of vaccine: generic, meningococcal, influenza, hexavalent, COVID-19, vaccination in pregnancy, other
- Population target of the vaccine: not specified, children, pregnant women, adults, elderly
- Source of information: mainstream media, social media native posts, no-source, other
- Kind of user: lay user, health professional (including medical doctors, nurses, physiotherapists and public health professionals, if indicated in the profile's bio), media companies and individual journalists, association (as specified in the profile's bio), institution (e.g., local health units, region, Ministry of Health, National Health Institute, WHO), other

For the qualitative analysis of people expressing discouraging and ambiguous stances, we studied the following topics identified through content analysis: efficacy, safety and conspiracy.

Training sessions and weekly meetings were organized by the study coordinators and followed by all involved researchers to share common rules and resolve doubts collegially.

Each tweet was classified by one researcher for all categories, except for the vaccine stance, which was classified by three researchers. Discrepancies in the stance classification were discussed together with a fourth researcher and a final stance classification was achieved collegially. Comparing the final agreed coding after discussions with the annotators' original initial coding, the accuracy of the individual annotators were 83.4% (FG), 80.6% (LP), and 68.8% (AP). If the tweet was a reply, researchers were allowed to access the tweet's URL to better understand the context. The study was conducted in line with the Ethical Guidelines 2.0 and 3.0 provided by the Association of Internet Researchers (26, 27). Considering the nature of the analysis, the current study did not require the approval of the local ethics committee according to current legislation, but a notification was sent to the Bambino Gesù Children's Hospital Ethical Committee. Only publicly available tweets were included in the study.

### Statistical Analysis

All variables were tabulated as frequencies and valid percentages and each was described by time period: pre-pandemic (before February 21, 2020) and during the pandemic (after February 21, 2020), taking into account that 21 February was the date in which the first native Italian COVID-19 case was detected. We used Multiple Correspondence Analysis (MCA) to identify variables associated with vaccine stance. The following variables were used to build the graph (“active” variables): tone of voice, stance, kind of author, information sources, kind of vaccine, and target population.

Data analysis was carried out using STATA V.14.1 SE (StataCorp, College Station, Texas, USA).

## Results

From an initial database of 3,187 randomly selected tweets, 714 tweets (22.40%) were excluded from the analysis. The majority of the excluded tweets were either unavailable at the time of the classification (e.g., they had been posted from an account that was canceled thereafter), had been posted by a private account, or had included the filter keywords only in a mentioned tweet or article and not in the actual text of the selected tweet. Other tweets were excluded as they were not in Italian (12/3,187, 0.38%), used the word vaccine in a figurative way (e.g., “there is no vaccine against ignorance”, 104/3,187, 3.26%) OR included information on the vaccination status of pets to be adopted or on cow's milk (in Italian, “latte vaccino”) (37/3,187, 1.16%).

The number of vaccine-related tweets identified by our system progressively increased from the beginning of the study period, reaching a peak in mid-April 2020. A temporary reduction of vaccine-related tweets is observed between January and February 2020.

Table 2 reports the characteristics of analyzed tweets by period (pre-pandemic vs. the first phase of the pandemic). Table 3 reports the tweet characteristics by stance. Supplementary Table 1 reports the characteristics of analyzed tweets by kind of vaccine.

**Table 2:** Characteristics of tweets by period (before and after 21 February 2020).

	**Pre-COVID-19** **(*****n*** **= 701)**		**During COVID-19** **(*****n*** **= 1,772)**		**Total** **(*****n*** **= 2,473)**	
	***n***	**%**	***n***	**%**	***n***	**%**
**Month**						
November 2019	169	24.1%	0	0.0%	169	6.8%
December 2019	235	33.5%	0	0.0%	235	9.5%
January 2020	237	33.8%	0	0.0%	237	9.6%
February 2020	60	8.6%	111	6.3%	171	6.9%
March 2020	0	0.0%	404	22.8%	404	16.3%
April 2020	0	0.0%	612	34.5%	612	24.7%
May 2020	0	0.0%	359	20.3%	359	14.5%
June 2020	0	0.0%	286	16.1%	286	11.6%
**Kind of vaccine**						
Generic	214	30.7%	237	13.4%	451	18.3%
Meningitis	100	14.3%	11	0.6%	111	4.5%
Influenza	97	13.9%	76	4.3%	173	7.0%
Vaccine in pregnancy	5	0.7%	0	-	5	0.2%
Hexavalent	57	8.2%	9	0.5%	66	2.7%
COVID-19	46	6.6%	1,388	79.6%	1,434	58.2%
MMR	65	9.3%	4	0.2%	69	2.8%
Other	114	16.3%	40	2.3%	154	6.3%
**Population target**						
Children	234	33.5%	20	1.1%	254	10.3%
Adult	84	12.0%	30	1.7%	114	4.6%
No population target	380	54.4%	1,715	97.2%	2,095	85.1%
**Stance**						
Promotional	118	16.8%	685	38.7%	803	32.5%
Ambiguous	6	0.9%	56	3.2%	62	2.5%
Discouraging	488	69.6%	455	25.7%	943	38.1%
Neutral	72	10.3%	478	27.0%	550	22.2%
Other	17	2.4%	98	5.5%	115	4.7%
**Tone of voice**						
Ironic	38	5.5%	151	8.6%	189	7.7%
Polemical/complaining	442	63.5%	699	39.6%	1,141	46.4%
Worried	51	7.3%	165	9.4%	216	8.8%
Neutral	83	11.9%	504	28.6%	587	23.9%
Other	82	11.8%	245	13.9%	327	13.3%
**Kind of author**						
Lay users	556	83.9%	1,298	80.5%	1,854	81.5%
Media companies or single journalist	45	6.8%	228	14.1%	273	12.0%
Other	62	9.4%	87	5.4%	149	6.5%
**Information source**						
Content published by mainstream media	190	27.6%	362	20.6%	552	22.6%
Social media posts	160	23.3%	34	1.9%	194	7.9%
Other	165	24.0%	181	10.3%	346	14.1%
No source	173	25.2%	1,182	67.2%	1,355	55.4%

**Table 3:** Characteristics of tweets by stance.

	**Stance**											
	**Promotional** **(*****n*** **= 803)**		**Ambiguous** **(*****n*** **= 62)**		**Discouraging** **(*****n*** **= 943)**		**Neutral** **(*****n*** **= 550)**		**Other** **(*****n*** **= 115)**		**Total** **(*****n*** **= 2,473)**	
	***n***	**%**	***n***	**%**	***n***	**%**	***n***	**%**	***n***	**%**	***n***	**%**
**Month**												
November 2019	36	4.5%	0	^*^	111	11.8%	20	3.6%	2	1.7%	169	6.8%
December 2019	13	1.6%	2	3.2%	206	21.9%	10	1.8%	4	3.5%	235	9.5%
January 2020	44	5.5%	4	6.5%	164	17.4%	19	3.5%	6	5.2%	237	9.6%
February 2020	79	9.8%	0	–	24	2.6%	55	10.0%	13	11.3%	171	6.9%
March 2020	172	21.4%	3	4.8%	61	6.5%	141	25.6%	27	23.5%	404	16.3%
April 2020	258	32.1%	21	33.9%	155	16.4%	146	26.6%	32	27.3%	612	24.8%
May 2020	107	13.3%	18	29.0%	127	13.5%	92	16.7%	15	13.0%	359	14.5%
June 2020	94	11.7%	14	22.6%	95	10.1%	67	12.2%	15	13.9%	286	11.6%
**Kind of vaccine**												
Generic	142	17.8%	3	4.8%	237	25.2%	41	7.5%	28	24.8%	451	18.3%
Meningitis	28	3.5%	1	1.6%	68	7.2%	13	2.4%	1	0.9%	111	4.5%
Influenza	53	6.6%	10	16.1%	91	9.7%	14	2.6%	5	4.4%	173	7.0%
Vaccine in pregnancy	0	0.0%	0	0.0%	5	0.5%	0	0.0%	0	0.0%	5	0.2%
Hexavalent	7	0.9%	2	3.2%	52	5.5%	2	0.4%	3	2.7%	66	2.7%
COVID-19	533	66.7%	40	64.5%	351	37.3%	443	80.7%	67	59.3%	1,434	58.2%
MMR	4	0.5%	2	3.2%	40	4.3%	20	3.6%	3	2.7%	69	2.8%
Other	32	4.0%	4	6.5%	96	10.2%	16	2.9%	6	5.3%	154	6.3%
**Population target**												
Children	33	4.1%	3	4.8%	181	19.3%	32	5.8%	5	4.4%	254	10.3%
Adult	36	4.5%	3	4.8%	61	6.5%	11	2.0%	3	2.7%	114	4.6%
No population target	730	91.4%	56	90.3%	697	74.2%	507	92.2%	105	92.9%	2,095	85.1%
**Tone of voice**												
Ironic	104	13.0%	2	3.2%	36	3.8%	29	5.3%	18	16.2%	189	7.7%
Polemical/complaining	249	31.2%	13	21.0%	708	75.5%	120	21.8%	51	45.9%	1,141	46.4%
Worried	89	11.1%	12	19.4%	69	7.4%	39	7.1%	7	6.3%	216	8.8%
Neutral	187	23.4%	11	17.7%	77	8.2%	299	54.4%	13	11.7%	587	23.9%
Other	170	21.3%	24	38.7%	48	5.1%	63	11.5%	22	19.8%	327	13.3%
**Kind of author**												
Lay users	596	79.9%	49	89.1%	814	93.0%	305	60.5%	90	93.8%	1,854	81.5%
Media companies or single journalist	94	12.6%	3	5.5%	19	2.2%	156	31.0%	1	1.0%	273	12.0%
Other	56	7.5%	3	5.5%	42	4.8%	43	8.5%	5	5.2%	149	6.5%
**Information source**												
Content published by mainstream media	133	16.7%	7	11.3%	175	18.8%	228	41.5%	9	8.2%	552	22.6%
Social media posts	12	1.5%	1	1.6%	166	17.8%	9	1.6%	6	5.5%	194	7.9%
Other	48	6.0%	1	1.6%	206	22.1%	83	15.1%	8	7.3%	346	14.1%
No source	602	75.7%	53	85.5%	384	41.2%	229	41.7%	87	79.1%	1,355	55.4%

### Kind of Vaccine and Population Target

**Figure 1:**
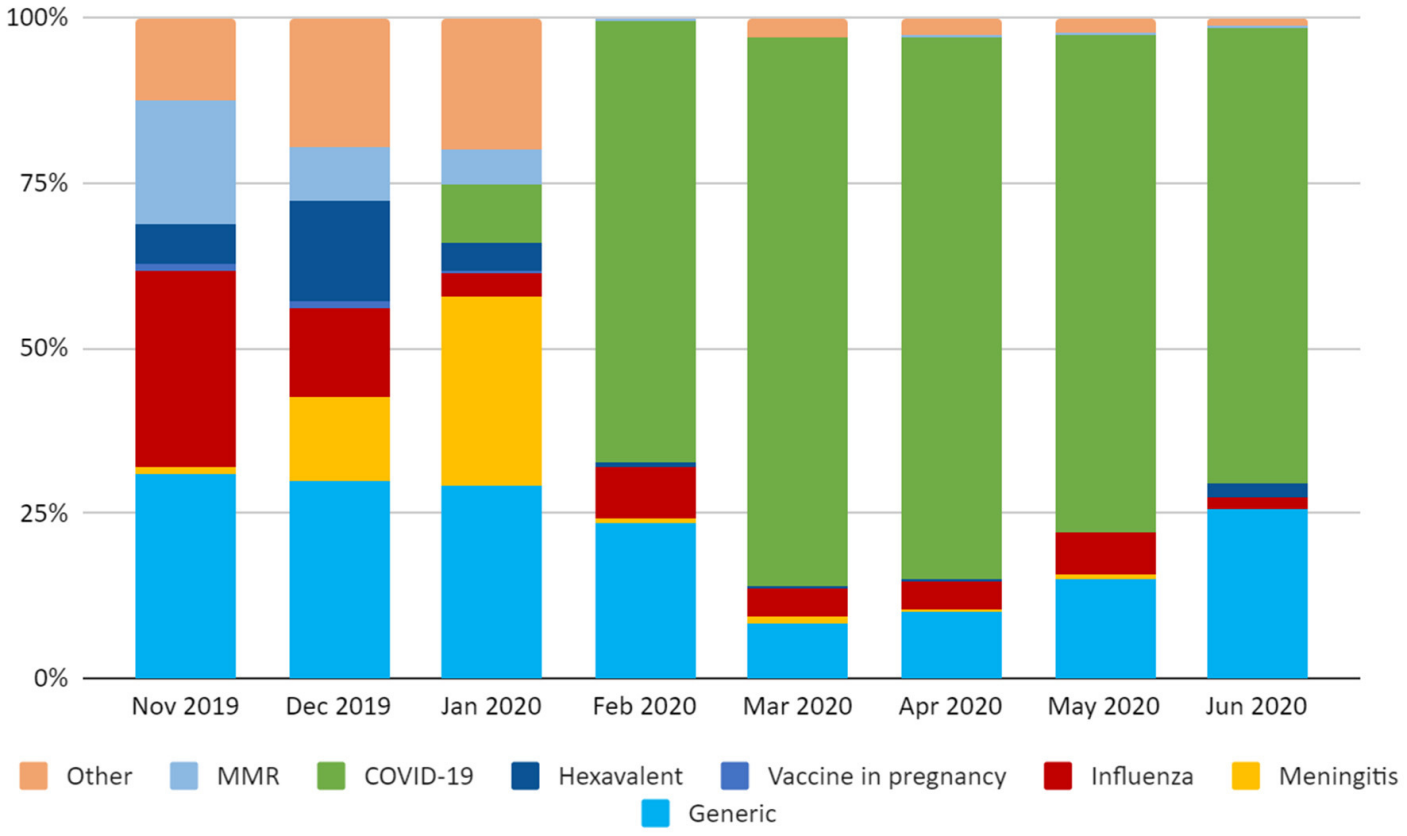
Monthly distribution of the kind of vaccine mentioned in the tweets in the study period.

The monthly variation of the kind of vaccine mentioned in the tweets is reported in Figure 1.

Overall, during the whole study period, 58.2% of downloaded tweets regarded the COVID-19 vaccine (1,434/2,463), despite our data being collected before the availability of COVID-19 vaccines. Some tweets (451/2,463, 18.3%) referred to vaccines generically, without mentioning a specific kind of immunization. Other vaccines mentioned in the tweets were the influenza vaccine (173/2,463, 7.0%), the vaccines against meningitis (111/2,463, 4.5%), the MMR vaccine (69/2,463, 2.8%), the hexavalent vaccine (66/2,463, 2.7%), and vaccines during pregnancy (5/2,463, 0.2%).

Seventy-six percent of the tweets referring to vaccines other than COVID-19 were posted before the pandemic. Among the vaccine-related tweets posted between November 2019 and February 21, 2020, those that referred to a specific kind of vaccine mentioned meningococcal vaccines (100/698, 14.3%), influenza (97/698, 13.9%), measles or MMR vaccine (65/698, 9.3%), hexavalent vaccine (57/698, 8.2%) and COVID-19 vaccine (46/698, 6.6%). The latter started to appear in Italian tweets in February 2020.

Between February-March and June 2020, the Twitter conversation was completely taken over by the COVID-19 vaccine, with a peak of 83.1% of COVID-19 related tweets over the total tweets published in March 2020. In this period, the other vaccines almost disappeared from the conversation, with the exception of the influenza vaccine, with a monthly proportion that ranged from 1.8% in June to 7.9% in February.

Most of the tweets during this period referred to vaccines without specifying a population target (2,095/2,463, 85.1%), and only 10% referred to childhood vaccinations. On the contrary, before the pandemic, one third of the conversation was focused on childhood vaccinations. The proportion of tweets explicitly mentioning other age categories was negligible (4.6% of the total, including adults, elderly and pregnant women) and were mainly posted before the pandemic.

### Stance

During the whole study period, the discouraging stance was the one most represented (943/2,473, 38.1%), 32.5% (803/2,473) were classified as promotional, mainly expressing trust toward science and vaccines or publishing scientific news, 22% had a neutral stance (550/2,473), while ambiguous tweets were the least represented (62/2,473, 2.5%). For 115 tweets, the stance was not understandable (4.7%). One third of the promotional tweets (270/803, 32.5%) were critical or sarcastic toward the no-vax community. Before the pandemic, the conversation in our random sample of tweets was dominated by the discouraging stance: before 21 February 2020, 69.6% (488/701) of the analyzed tweets had a discouraging stance, while promotional and neutral tweets represented a very low proportion of the total tweets (16.8 and 10.3%, respectively).

**Figure 2:**
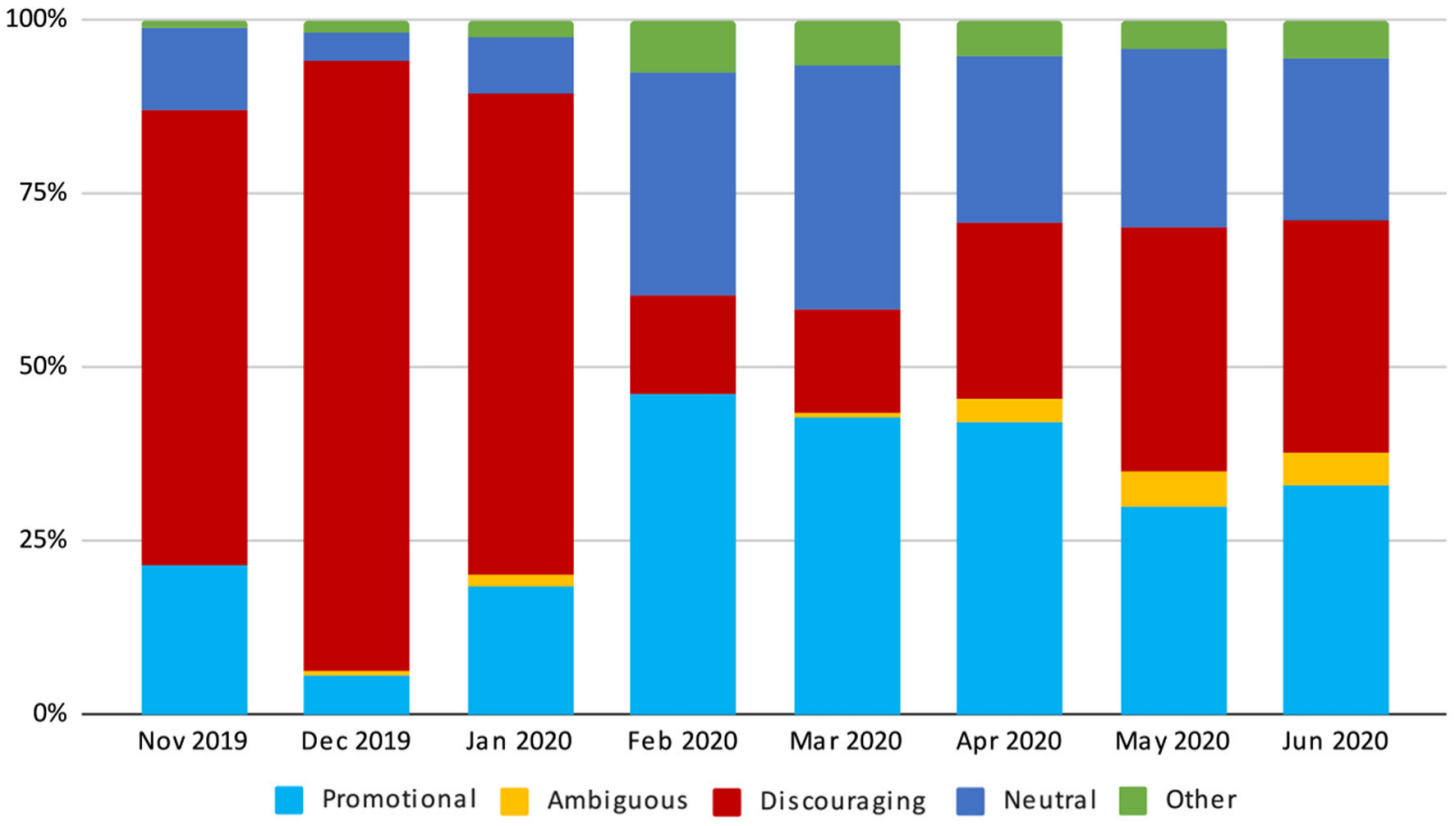
Monthly distribution of the tweets' vaccine stance in the study period.

Looking at the monthly data (see Figure 2), an evident change in the distribution of the stance was detected in February and March 2020. In these 2 months, discouraging tweets decreased intensely, both in absolute numbers (from 6.5 discouraging daily tweets as detected by our system between November 2019 and January 2020 to 1.4 discouraging daily tweets between February and March 2020) and proportionally to other stances (from 75.0% before February to 14.7% in February and March). In February and March, the vaccine discourse was dominated by promotional tweets (251/575, 43%), followed by neutral tweets (196/575, 34.0%).

Between April and June 2020, the proportions of tweets were more stable. Promotional tweets still had a prominent role in the vaccine conversation, though with a lower proportion compared to the two previous months (459/1,257, 36.5%). Discouraging tweets increased again, though not reaching the pre-pandemic levels (377/1,257, 30.0%), and had a larger volume compared to neutral tweets (305/1,257, 24.3%).

The number of ambiguous tweets was negligible before and during the first months of the pandemic (we recorded only 9 tweets between November 2019 and March 2020, corresponding to 0.7% of the total tweets in the same period), but started increasing between April and May 2020, although remaining a very limited proportion of the total tweets (53/1,257, 4.22%).

We also analyzed how the stance varied in relation to the type of vaccine mentioned. Among tweets mentioning non-COVID-19 vaccines, the most represented stances were the following: discouraging (57.2%), promotional (25.9%) and neutral (10.3%). On the other hand, when considering COVID-19 vaccines only, the stance varied as follows: promotional (37.2%), neutral (30.9%) and discouraging (24.5%).

### Tone of Voice

**Figure 3:**
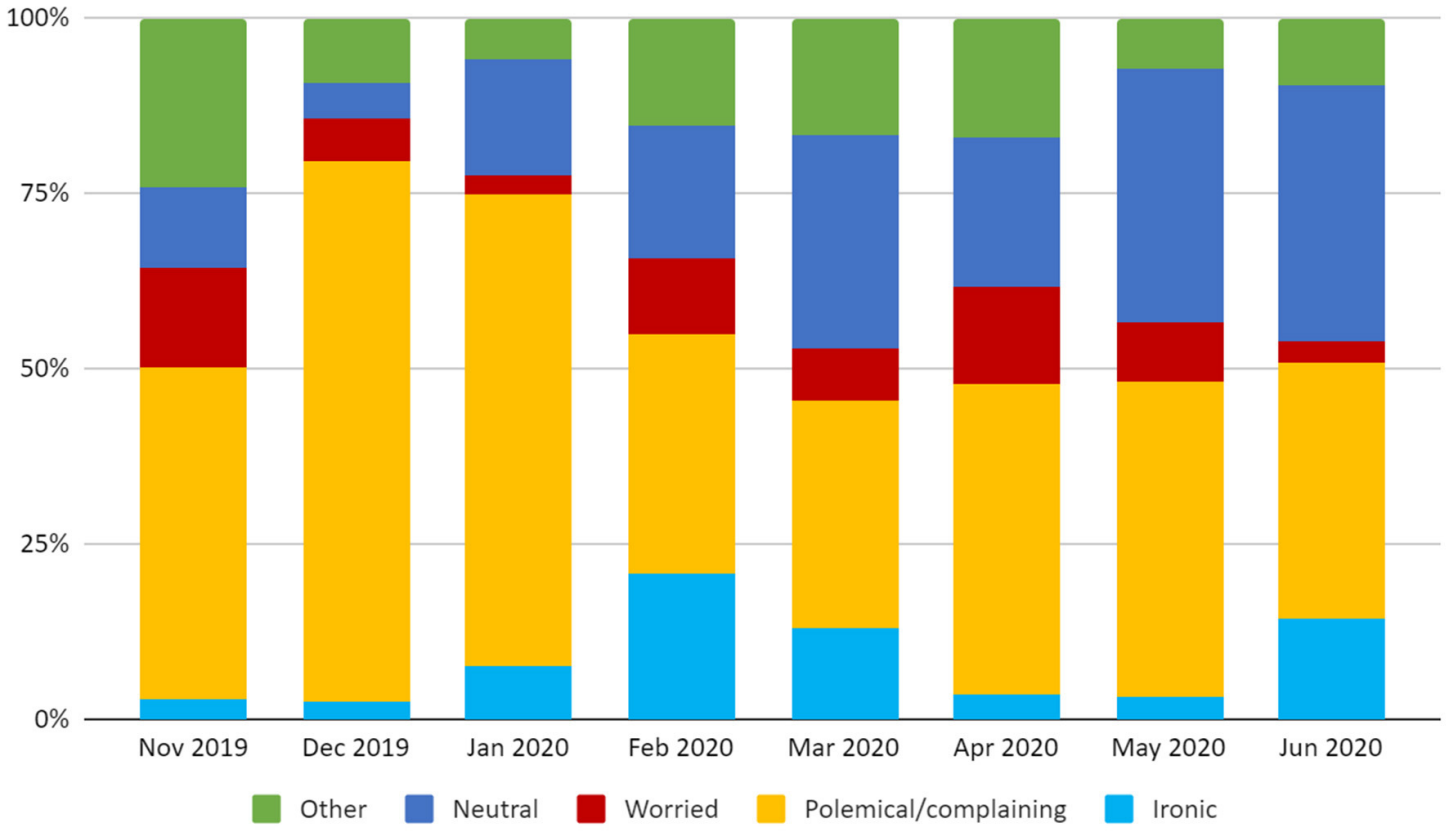
Monthly distribution of the tweets' tone of voice in the study period.

Almost half of the downloaded tweets had a polemical/complaining tone of voice (1,141/2,473, 46.4%), followed by neutral (587/2,473, 23.9%), worried (216/2,473, 8.8%) and ironic (189/2,473, 7.7%). Tones of voice with a more positive nuance were the least represented (purposeful 105/2,473, 4.2%, supportive/empathic 31/2,473, 1.3%, enthusiastic 53/2,473, 2.1%), and so were tweets with an interrogative tone of voice (104/2,473, 4.2%). Tweets classified as “other” for the tone of voice category were 34 (1.4%). For the purpose of the analysis, these last 5 tones of voice were grouped in a single category (“Other”, 13.3%). Different stances are associated with specific tones of voice: 75.5% of the discouraging tweets are polemical/complaining; an interrogative tone of voice characterizes several ambiguous tweets (32.3%); promotional tweets (including tweets against no-vax) are generally using a polemic/complaining (31.2%) or neutral (23.4%) tone of voice. The monthly variation of the tone of voice is reported in Figure 3.

### Kind of Author and Information Source

The large majority of authors were lay users (1,854/2,276, 81.5%). Twelve percent of the tweets (273/2,276) were posted by media companies or by individual journalists. Only 1.5% were posted by health care professionals, and 0.1% by institutions. The remaining 5% of tweets were posted by associations and other kinds of sources.

More than half of the tweets did not mention any source of information (1,355/2,447, 55.4%). The most frequently mentioned source of information was content published by mainstream media (552/2,447, 22.6%), followed by social media posts (194/2,447, 7.9%) and blog/website/forums (176/2,447, 7.2%). Alternative medicine sources were mentioned in 2.1% of tweets (52/2,447), while institutional contents were mentioned only in 2.4% of the tweets (58/2,447).

The emergence of the COVID-19 crisis affected the use of sources for information reported in the tweets. Before the COVID-19 crisis, tweets reported information from mainstream media (190/688, 27.6%) or other social media (160/688, 23.3%), with only one fourth of the tweets reporting no source of information. After February 21, 202, Twitter became a more instinctual and reactive arena, with the great majority of the tweets reporting no information source (1,182/1,759, 67.2%).

### Stance Profiles

**Figure 4:**
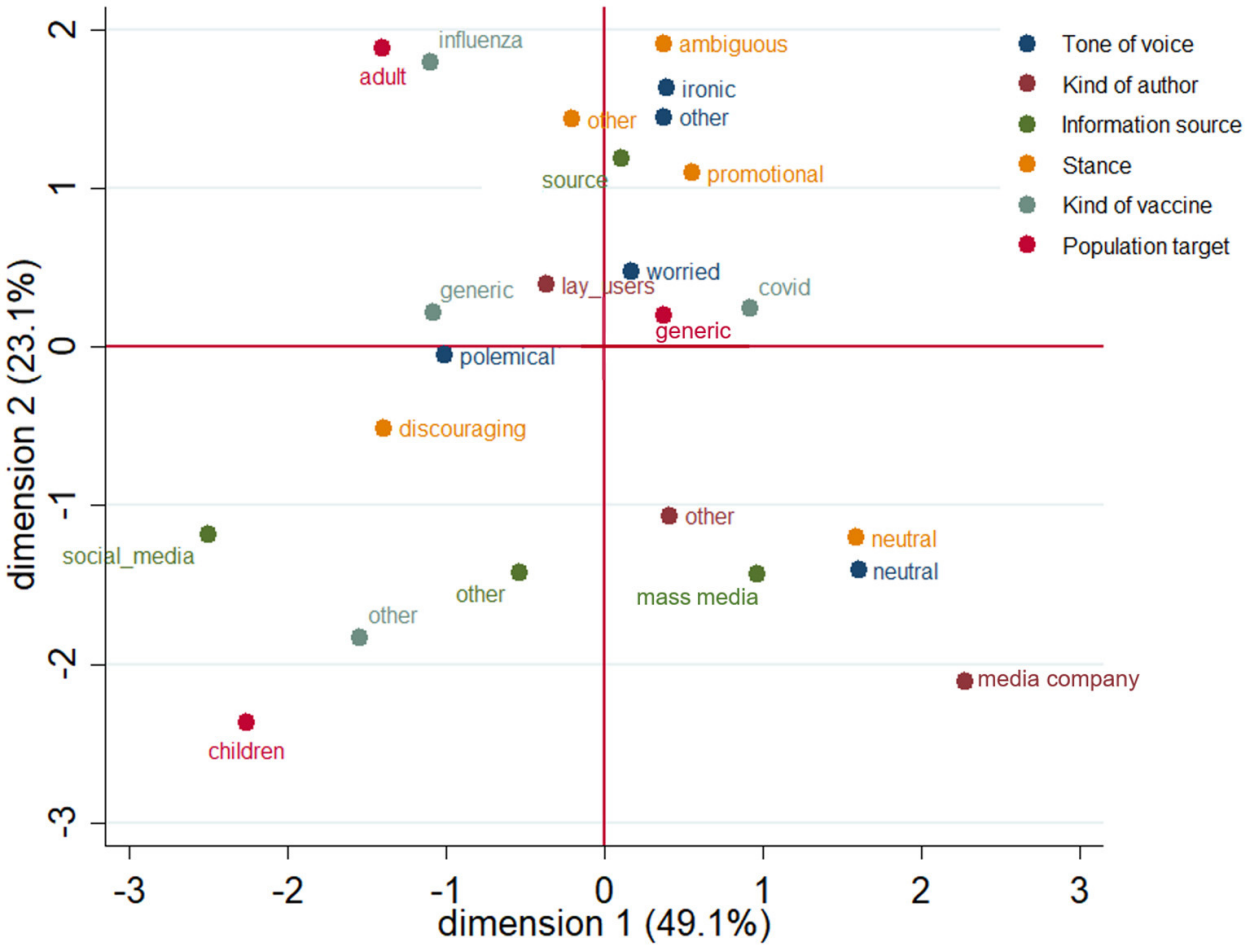
Multiple corresponding analysis.

We analyzed stance profiles in order to identify how promotional, ambiguous, discouraging and neutral tweets were associated with other variables. According to the multiple correspondence analysis, the results of which are mapped in Figure 4, the tweets with a neutral and a discouraging stance were clearly associated to specific characteristics, which enabled us to identify relatively definite profiles.

Neutral tweets were more associated with a neutral tone of voice, were more frequently posted by media accounts and mainly mentioned mainstream media as information sources.

Discouraging tweets had a polemical/complaining tone of voice, usually did not refer to specific vaccines and often reported other social media posts as source of information. We also observed a trend of association of tweets regarding vaccines targeting children with a discouraging stance. Indeed, the great majority of the tweets addressing children, elderly and pregnant women were discouraging (respectively, children, 71%; elderly 73%; and pregnant women, 100%).

On the other hand, promotional and ambiguous tweets did not show a definite segregation on the graph, therefore we can assume that these stances were not associated with definite characteristics.

### Qualitative Analysis of People Expressing Discouraging and Ambiguous Stance

To better understand users' motivations and beliefs related to vaccines, we carried out a qualitative analysis of tweets expressing discouraging and ambiguous stances. The qualitative analysis revealed the macro dimensions describing discouraging and ambiguous tweets and how they changed before and during the COVID-19 crisis. Users producing discouraging or ambiguous tweets referred to the following main issues: efficacy, safety, conspiracy and other topics. Some interesting differences emerge between discouraging and ambiguous tweets.

On the discouraging side, almost all the negative issues about vaccines were raised by lay users and were related to three main topics: conspiracy (46.7%); safety (37.5%); and efficacy (11.8%). Discouraging tweets referring to conspiracy generally do not include any source of information (53%, 268/508). A typical reasoning is the following: “Big Pharma” creates viruses in labs in order to test drugs and increase their profits selling vaccines; in the meantime, governments and scientists are part of this plot and use viruses, including SARS-CoV-2, to legitimize the control on citizens' behaviors or even destroy humanity. The following three tweets refer to different versions of this approach.

“Cari genitori IGNORANTI, non avete più scuse! Salvate i vs. figli da questa carneficina, le informazioni ci sono e questa la prova! #Lorenzin &co hanno venduto i ns. figli alle #Bigpharma per testare i #vaccini, CAVIE null'altro che cavie da laboratorio” (24th November, 2019).

*[Dear IGNORANT parents, you have no more excuses! Save your children from this carnage, the information is there and this is the proof! #Lorenzin [a former Italian's Ministry of Health] & co have sold our children to #Bigpharma to test #vaccines, GUINEA PIGS nothing but laboratory guinea pigs.]*

“l'ultimo pezzo dell'articolo avvalora la tesi della dottoressa americana che ha denunciato tutto,il vaccino serve a sviluppare una risposta immunitaria cosi potente che al contatto col virus selvaggio uccide chi lo ha ricevuto, se lo ammettono loro immaginate cosa nascondono!” (28th May, 2020).

*[The last piece of the article confirms the theory of the American doctor who exposed everything, the vaccine is used to develop an immune response so powerful that when it comes into contact with the wild virus it kills those who received it, if they admit this, imagine what else they are hiding!]*

“Siete contenti? Il vostro presidente del consiglio ha trovato il vaccino ed ha trovato anche i soldi..SVEGLIA! A QUESTI DI NOI NON INTERESSA NIENTE!” (13th June, 2020).

*[Are you happy? Your prime minister found the vaccine and he found the money too. WAKE UP! THEY DO NOT CARE ABOUT US!]*

Conspiracy narratives are variable; however, they all express distrust against institutions and offer hyper-simplified plots, based on alternative truths revealing the hidden interests of the elites. Discouraging tweets referring to safety do not generally focus on COVID-19 vaccines; moreover, assumptions about safety issues are very broad and sources are heterogeneous (no source, mass media, social media posts). Most popular statements claim that vaccines contain dangerous components (e.g., fetal cells, mercury) produce severe side effects, generate victims among fragile people (elderly and kids) and cause diseases (e.g., autism).

“Considerando ke ogni vaccino contiene la quantita' di mercurio,alluminio e formaldeide 100 volte massima consentita,fare il calcolo.Lo stesso vale per i vaccini obbligatori per i bambini.da qui l'autismo,asma,malattie immunitarie e neurodegenerative in seguito.INFORMATEVI prima!” (17th November, 2019).

*[Considering that each vaccine contains the maximum amount of mercury, aluminum and formaldehyde 100 times the maximum allowed, do the math. The same goes for mandatory vaccines for children, hence autism, asthma, immune and neurodegenerative diseases later. INFORM YOURSELVES first!]*

Half of the discouraging tweets regarding efficacy are related to COVID-19 (50.7%, 68/134) and do not add any source to their post (62.7%, 84/134). Most of these users believe vaccines are not effective and cannot prevent the spread of epidemics.

“Sono mesi che medici seri dicono che il VACCINO NON SERVE perché non funzionerebbe! Però mass media e politici continuano a dire che lo aspettano! Posso immaginare perché” (26th May, 2020).

*[Serious doctors have been saying for months that the VACCINE IS NOT USEFUL because it would not work! But the mass media and politicians continue to say they are waiting for it! I can imagine why.]*

Most of the ambiguous tweets were produced after the beginning of the COVID-19 crisis (50 out of 62). A close reading of the concerns people brought up revealed genuine questions and doubts. Talking about vaccines in general, some users stated they would have liked to receive more information about side effects before receiving the vaccination, others looked for more information about pharmacovigilance data reporting vaccine side effects; some other people questioned the Italian vaccine mandate. With regards to the COVID-19 vaccine, several users were interested in better understanding vaccine efficacy mechanisms, for example among people who already got the virus:

“Dicono che per il Covid serva vaccinarsi. Chi lo ha già avuto perché dovrebbe fare il vaccino? Quanto dura eventualmente l'immunità? Se averlo avuto non immunizza c^****^ serve il vaccino? Qualcuno sa qualcosa?” (19th April, 2020).

*[They say that for Covid you need to get vaccinated. Those who have already had it, why should they get the vaccine? How long does immunity eventually last? If having had the virus doesn't immunize you, why the f* ^***^ *do you need a vaccine? Anyone know anything?]*

Some people expressed doubts on the relationship between the influenza vaccine and the SARS-CoV-2 virus wanting to understand if the influenza vaccine could protect from, or even be a cause of, COVID-19.

“Premetto che faccio il vaccino antinfluenzale volontariamente da un paio d'anni. Ma prima che sia reso obbligatorio, cosa cmq molto illiberale, sarebbe doverosa una statistica che non ho mai letto: quale percentuale nel contagio coronavirus tra vaccinati contro influenza e no*?*” (16th May, 2020).

*[I admit that I have voluntarily been receiving the flu vaccine for a few years. Before it becomes obligatory, which would be very illiberal, we need a statistic that I have not yet seen: what is the percentage of coronavirus contagion amongst those vaccinated against the flu?]*

Similarly to discouraging tweets, some hesitant tweets expressed skepticism about the speed of production of the COVID-19 vaccine.

“Non sono contro i vaccini e non sono stupida. Ma se mi dite che ci vogliono 18 mesi per un vaccino e poi me lo proponete a ottobre, due domandine me le faccio” (21st June, 2020).

*[I'm not against vaccines and I'm not stupid. But if you tell me that it takes 18 months to make a vaccine and then you offer it to me in October, I have a few questions.]*

## Discussion

In the present article, we show how the Italian Twitter conversation on vaccines changed during the first phase of the COVID-19 pandemic, compared to the immediate pre-pandemic period. We performed a detailed quantitative and qualitative analysis of a corpus of tweets, which provided us with an in-depth understanding of the discourse on vaccines on Twitter in Italy and of the actors involved in it.

The first striking result was the high representation of discouraging tweets in our sample. Overall, discouraging tweets represented almost 40% of the whole corpus. In the pre-pandemic period, based on our results, discouraging tweets dominated the vaccine conversation on Twitter, with an average frequency of 75% and a peak of 86% in December 2019. The focus of the tweets in this period was mainly on vaccines against meningitis, influenza and MMR.

At the beginning of the COVID-19 crisis, in February and March 2020, the conversation was quickly catalyzed by the COVID-19 vaccine, and a large part of the users expressing a discouraging stance seemed to silence their voice, with the proportion of discouraging tweets decreasing to 15%. In parallel, we have observed a striking increase in neutral tweets and in the involvement of users with a promotional stance from February 2020, when COVID-19 became the main news reported in any media. We hypothesize that this striking change at the beginning of the pandemic does not reflect a change in vaccine confidence among Twitter users, but rather a change of users' engagement in the vaccine discourse, which can be explained by different communication theories. On one hand, the observed change seems to reproduce a sort of “spiral of silence”, which refers to the well-known communication theory (28) claiming that people tend not to express their opinion on a public issue when they feel they have a minority viewpoint, whereas people perceiving themselves as part of a majority are more inclined to speak out. Following this model, at the very beginning of the COVID-19 pandemic, anti-vax users in Italy may have decreased their activity on Twitter because they were scared by the virus and the risks of the pandemic, or perceived that criticizing vaccines during such a severe crisis was not appropriate or convenient. On the other hand, the observed increase of neutral and promotional tweets is certainly driven by agenda setting routines, mainly emphasizing the production of the COVID-19 vaccine, which reflect the ability of the news media to influence the importance of the topics on the public agenda (29). According to the agenda setting theory, (social) media conversations shape and are shaped by agenda setting patterns, i.e., contents produced by social media users reflect most popular content circulating on news media - and vice-versa. In this context, lay users seem to voice their opinion on Twitter only if triggered by a significant media event. This behavior is actually in line with the nature of Twitter, where the conversation is algorithmically driven by trending topics highlighting the most emerging themes, thus miming human interest for newness.

From April 2020, discouraging tweets seem to regain more weight in the Twitter conversation (representing almost one third of the tweets), though still not reaching pre-pandemic levels (69% discouraging tweets in January vs. 25% in April 2020), at least until June 2020. In parallel, promotional and neutral tweets kept being more represented compared to the pre-pandemic levels. In this phase, Twitter users may have perceived that the COVID-19 vaccine could have offered an actual solution for overcoming the crisis—a prevalent narrative in these months was “the COVID-19 vaccine is the light at the end of the tunnel”. Given the polarization of the Twitter debate on vaccines, this scenario may have stimulated more reactions from discouraging users, among which also conspiracy theories on the origin of the pandemic and on the COVID-19 vaccine started to regain popularity together with already “established” narratives questioning the vaccines' efficacy and safety.

Moreover, with the beginning of the COVID-19 crisis, other vaccines that were previously addressed with a prevalent discouraging stance (such as meningococcal, hexavalent, and MMR), almost disappeared from the Twitter conversation, which turned into a mainly COVID-19 vaccine-related conversation. The decrease in the conversation on other vaccines might reflect a decrease in interest in this topic by the general public, which might have had an impact on vaccine coverage. Between April and June 2020 (30), according to a recent survey (31), a worrying trend of routine vaccination delays was observed in Italy, likely driven by fears of contact with COVID-19 cases at vaccination centers, issues with vaccination centers' logistics and lack of information. Italian data are in line with trends seen at the global level (32, 33). This highlights the important role of public health and government in having a social media presence and in communicating the importance of routine vaccinations in preventing the potential emergence of additional epidemics during emergency situations.

Another interesting result is the very low representation of ambiguous tweets—especially in the pre-pandemic phase. We applied the stance classification previously used by the Vaccine Confidence Project (VCP) for its consistency, reproducibility and because it allowed us to better compare our results with other countries. Nevertheless, the proportion of ambiguous tweets on the total tweet corpus, though slightly increasing after the beginning of the pandemic, is almost negligible. This is probably due to Twitter affordances and prevailing usage practices among Italian users (prevalence of public profiles and interest to get visibility in the public debate): indeed, Italian Twitter users tend to express very definite opinions rather than sharing their uncertainties. This is confirmed by other studies clearly showing a strong polarization of social media communities around controversial topics, including the vaccination debate (34, 35).

However, hesitant, undecided users, although their beliefs are less visible, are actually exposed to tweets expressing polarized opinions (promotional and discouraging tweets) (36). In other words, polarized tweets influence the climate of opinion and may even contribute to reducing the willingness of hesitant users to speak out. In the previously mentioned study by the VCP, Italian tweets analyzed in terms of stance, although limited in number, showed a proportion of ambiguous tweets of 16.4%. Nevertheless, the study specifically focused on vaccination in pregnant women, which might have elicited a higher level of uncertainty among Twitter users compared to vaccines targeting other groups. In our tweet corpus, most of the ambiguous tweets were produced after the beginning of the pandemic. This shows that in the first phase of the pandemic, before the actual production of the vaccine, news related to the production of a new vaccine creates more room to express questions and doubts. We cannot exclude the possibility that, in the months following our study, a higher representation of ambiguous tweets has been elicited by rumors regarding the COVID-19 vaccine and by news on adverse events reported in the general media, during the COVID-19 vaccination campaign.

Overall, previous analyses on the Italian vaccine conversation show a high variability of stance/sentiment distribution among the studied tweet corpuses. While the heterogeneity of methods and objectives of these researches make it difficult to perform comparisons, it is interesting to note how the context has a strong influence on the stance/sentiment distribution.

In 2016, we analyzed tweets posted with regards to two broadcasts dedicated to vaccines aired on Italian television, showing a prevalence of 50% of what they defined as “positive” sentiment, and a very low proportion of negative sentiment (10%) (37). An analysis carried out with text mining techniques on Italian tweets published between September, 2016 and August, 2017, showed a prevalence of neutral tweets (60%), followed by tweets against vaccination (23%), while the proportion of tweets in favor was the lowest (17%) (38). Finally, Ajovalasit et al. (39) applied machine learning techniques to a corpus of vaccine-related Italian tweets posted in 2018, and identified 70% of tweets as favorable to vaccination, 16% as unfavorable and 14% as undecided. It is also interesting to note how the proportion of vaccine stances expressed in tweets might differ from that recorded through traditional surveys, on samples which are usually more representative of the Italian population than Twitter users. A survey investigating vaccine knowledge and attitudes in parents of young children, performed by the Italian National Institute of Health between 2015 and 2016 showed a very high majority of pro-vaccine parents (83.7%), a lower proportion of hesitant (15.6%) and proportion of anti-vaccine parents below 1% (40).

The heterogeneity of published data on vaccine hesitancy on Twitter and on the general population in Italy prompt the need of a coordinated action by institutions to set the basis for a recurrent monitoring of vaccine hesitancy in the population, to be performed both by traditional surveys and social media analysis. A systematic monitoring of this kind of data would enable us to obtain a clear and consistent picture of vaccine hesitancy in the population and to plan timely, data-based communication strategies.

Social media monitoring can also provide nuanced information on the tone of voice of the conversation and on the topics explored by users, which, in contrast to traditional surveys, have the advantage of being a kind of data spontaneously provided by social media users.

Tone of voice analysis revealed that the prevalent tone of voice in Twitter conversations on vaccines is polemical/complaining. This result confirms the observation that social media in Italy are considered as the grounds for vaccine controversy between two opponents (41), leaving no space for a more reasoned and non-partisan debate. This approach is confirmed by the overall prevalence of user-generated content (tweets citing no-source of information). However, the analysis revealed that the beginning of the COVID-19 crisis challenged the framing of the Twitter debate about vaccines, increasing the presence of promotional tweets with a neutral tone of voice and reducing the concerns related to vaccine safety.

A very important result that emerges from our study is the very low prevalence of tweets posted by institutional accounts. Out of 2,463 tweets, we found just one tweet by the Italian Ministry of Health promoting the influenza vaccine and one by the Lombardia Region reporting the link to an interview to an infectious disease specialist on COVID-19. Although it could be biased by the random sampling of the tweets, it suggests that Italian national and local health institutions have been scarcely visible in the vaccine conversation on Twitter during the initial phase of the pandemic and did not exert any relevant influence during the analyzed period. As Lovari recently described (42), during the COVID-19 crisis, the Italian Ministry of Health expanded its presence mainly on Facebook, even if the engagement of the MoH Facebook account in the conversation with users was very limited. This confirms a limited involvement of institutional social media accounts during previous natural disasters or national crises in Italy (43). An improvement of the engagement in the conversations with users has been recommended in the past to institutions and local health departments, based on the observation that communication by institutional social media accounts was mainly one-way, as opposed to a dialogue (44, 45). In the US, health departments are starting to adopt a more consistent social media strategy (46), and social media toolkits for health departments have been published (47). As a matter of fact, the need of a stronger presence on social media has been acknowledged by Italian institutions and has improved during the pandemic.

The multiple correspondence analysis allowed us to identify the variables associated with the neutral and the discouraging vaccine stance, and better describe tweets exhibiting either of these two stances: neutral tweets were posted by mainstream media Twitter profiles with a wide public, while discouraging tweets were associated with a complaining tone of voice and with the use of other social media posts as sources of information. The characteristics of tweets with either a promotional or ambiguous stance were more blurred and less specific.

More research would be useful to understand if the definite profile of discouraging users can be accounted as one of the reasons for their success on social media. As a matter of fact, social media platforms are interested in monitoring users' behaviors and selling their data to advertisers to make a profit (a concept called “platform-capitalism”), and they pursue this objective by increasing the amount of time users spend online through algorithms that affect the visibility of published content, following specific ordering criteria (namely, ranking). As a result, controversial topics (like vaccines), addressed with polemical/complaining tone of voice (the most frequent stance and tone of voice in our database) significantly contribute to catch users' attention and reactions, and thus enlarge platforms' profits. Therefore, we cannot exclude that the high circulation of this kind of polarized and conflictual content could be biased by a commercial interest of the platform. On the other hand, social media platforms, including Twitter, have reinforced their policies with regards to COVID-19 misinformation, prioritizing removal or annotation of potentially harmful and misleading information. More research is needed to understand the effect of such policies on polarization in the vaccination debate.

Our study has a number of limitations. The main limitation is that the analyzed tweet dataset was randomly extracted from a larger corpus of vaccine-related tweets; therefore the study may suffer from sampling bias due the small proportion of records compared to the total Italian vaccine-related Twitter conversation. Nevertheless, we conducted this complex assessment of Italian tweets on vaccines with the purpose of highlighting some trends and characteristics that could constitute a basis for future social media monitoring activities and communication initiatives. As the manual classification of tweets is highly consuming in terms of researchers' energy and time, the use of a machine learning-based algorithm for automatic stance classification would be advisable, as it would enable us to describe the stance of a larger tweet corpus and to describe the stance variation over time in a more reliable and unbiased way. Our results can also be useful to identify critical issues that could better guide the training of a machine-learning algorithm. Secondly, tweets were coded by three researchers in terms of stance, while the other categories were classified by one researcher only, which might have impaired the reliability of the classification. Nevertheless, all classifiers have been following training sessions and periodic meetings to refine the uniformity of the methods and common classification rules, in order to achieve a high level of agreement. Thirdly, the study is limited to June 2020, and the vaccine discourse might have radically changed in the following months, with the start of the vaccination campaigns. Moreover, Twitter policy about profiles spreading misinformation might also have changed the actors and the contents of the conversation on Twitter in the following months. Because of this change of policy, some tweets, although originally downloaded by our platform, were unavailable at the time of the analysis as they had been removed by Twitter after being published. This might imply that the amount of discouraging tweets could be slightly underestimated. Twitter in Italy is not the most popular social media (48) and it is mainly used as a public relation tool to comment on public events and news in real time. Therefore, the generalizability of our results to the general population is limited and would require the integration of the Twitter data with data from other social media.

Our study has several strengths. First, it deeply investigates characteristics and nuances of the Twitter conversation before and during the first months of a global emergency, through the analysis of a large corpus of tweets by researchers with different backgrounds (health, epidemiology, communication, internet studies). The issues highlighted in this paper can be useful for health communication specialists, as they could generally characterize the reaction of social media users to a health emergency. Moreover, our results set a baseline, which can be used for comparison in future studies on the Italian vaccination discourse, and which can therefore provide useful insights for vaccine communication.

## Conclusion and Implications

The paper highlighted how the emergence of a new virus (i.e., SARS-CoV-2) caused a deep change in the vaccination discourse on Twitter in Italy, limiting the vocality of discouraging tweets, increasing the presence of promotional tweets with a neutral tone of voice and slightly encouraging the emergence of ambiguous opinions about vaccines in the public debate on social media.

Our results have a number of implications.

First, we confirm that public institutions in Italy should continue to improve their presence on Twitter, engaging in conversation both with promotional users, who can amplify science based promotional messages, and with hesitant individuals, who, although relatively silent on Twitter, are exposed to the polarized vaccine-related conversation and would benefit from the exposure to reliable, science-based facts.

Secondly, we highlight a trend from which public institutions should benefit in an emergency context: listening and answering to the doubts and questions in a timely manner during the initial stage of a health crisis, before they turn into more radical discouraging assumptions or new conflicts. This could help in preventing vaccine hesitancy through the timely “inoculation” of science-based facts. For example, the qualitative analysis highlighted a fear (i.e., the speed of the COVID-19 vaccine production process), that appeared quite early in the COVID-19 vaccine debate and that has been subsequently confirmed as one important driver of vaccine hesitancy.

Third, the conversation on routine vaccinations should be supported and reinforced even if the conversation is catalyzed by a health crisis or by new vaccines, to avoid the reduction of vaccine coverage due to vaccination delays or hesitancy.

Finally, a more complete picture of vaccine stance in Italy would benefit from systematic social media monitoring, and integration of Twitter data with data from other social media platforms. Ideally, the information collected should be combined with traditional surveys performed on representative samples of the population. This could also enable us to better understand the relation between opinions expressed on social media and the actual vaccination attitude of the population, and, based on this data, plan tailored, data-based communication strategies.

## Data Availability Statement

The original contributions presented in the study are included in the article/Supplementary Material, further inquiries can be directed to the corresponding author/s.

## Author Contributions

FG, LP, FC, and CR contributed to the conception and design of the study and supervised the tweet classification. FG and LP drafted the manuscript. AP, LR, IC, and BL contributed to data management and tweet classification. IC performed the statistical analysis. MR, AF, AT, and CR reviewed the manuscript. All authors read and approved the submitted version.

## Funding

This study was funded by Grant No. 801495 of the Consumer, Health, Agriculture and Food Executive Agency (CHAFEA).

## Conflict of Interest

AT received grants from MSD for invited lectures. The remaining authors declare that the research was conducted in the absence of any commercial or financial relationships that could be construed as a potential conflict of interest. The reviewer CN declared a past collaboration with the authors FG, MR, AT, and CR to the handling editor.

## Publisher's Note

All claims expressed in this article are solely those of the authors and do not necessarily represent those of their affiliated organizations, or those of the publisher, the editors and the reviewers. Any product that may be evaluated in this article, or claim that may be made by its manufacturer, is not guaranteed or endorsed by the publisher.

## References

[1] European Centre for Disease Prevention and Control. Systematic Scoping Review on Social Media Monitoring Methods and Interventions Relating to Vaccine Hesitancy. LU: Publications Office (2019). Available online at: https://data.europa.eu/doi/10.2900/260624 (accessed October 27, 2021).

[2] Ford AJ Alwan NA. Use of social networking sites and women's decision to receive vaccinations during pregnancy: a cross-sectional study in the UK. Vaccine. (2018) 36:5294–303. 10.1016/j.vaccine.2018.07.022 30055969

[3] Hou Z Tong Y Du F Lu L Zhao S Yu K. Assessing COVID-19 vaccine hesitancy, confidence, and public engagement: a global social listening study. J Med Internet Res. (2021) 23:e27632. 10.2196/27632 34061757 PMC8202656

[4] Islam MS Kamal A-HM Kabir A Southern DL Khan SH Hasan SMM. COVID-19 vaccine rumors and conspiracy theories: the need for cognitive inoculation against misinformation to improve vaccine adherence. PLoS ONE. (2021) 16:e0251605. 10.1371/journal.pone.0251605 33979412 PMC8115834

[5] Mohanty S Leader AE Gibeau E Johnson C. Using Facebook to reach adolescents for human papillomavirus (HPV) vaccination. Vaccine. (2018) 36:5955–5961. 10.1016/j.vaccine.2018.08.060 30172634

[6] Cinelli M Quattrociocchi W Galeazzi A Valensise CM Brugnoli E Schmidt AL. The COVID-19 social media infodemic. Sci Rep. (2020) 10:16598. 10.1038/s41598-020-73510-5 33024152 PMC7538912

[7] Tangcharoensathien V Calleja N Nguyen T Purnat T D'Agostino M Garcia-Saiso S. Framework for managing the COVID-19 infodemic: methods and results of an online, crowdsourced WHO technical consultation. J Med Internet Res. (2020) 22:e19659. 10.2196/19659 32558655 PMC7332158

[8] Loomba S de Figueiredo A Piatek SJ de Graaf K Larson HJ. Measuring the impact of COVID-19 vaccine misinformation on vaccination intent in the UK and USA. Nat Hum Behav. (2021) 5:337–48. 10.1038/s41562-021-01056-1 33547453

[9] EU-JAV - European Joint Action on Vaccination. EU-JAV. Available online at: https://eu-jav.com/ (accessed October 27, 2021).

[10] D'Ancona F D'Amario C Maraglino F Rezza G Ricciardi W Iannazzo S. Introduction of new and reinforcement of existing compulsory vaccinations in Italy: first evaluation of the impact on vaccination coverage in 2017. Eurosurveillance. (2018) 23:1800238. 10.2807/1560-7917.ES.2018.23.22.1800238 29871721 PMC6152175

[11] Trova Norme & Concorsi - Normativa Sanitaria. Available online at: https://www.trovanorme.salute.gov.it/norme/dettaglioAtto?id=60201 (accessed October 27, 2021).

[12] Sabbatucci M Odone A Signorelli C Siddu A Maraglino F Rezza G. Improved temporal trends of vaccination coverage rates in childhood after the mandatory vaccination act, Italy 2014–2019. JCM. (2021) 10:2540. 10.3390/jcm10122540 34201199 PMC8230222

[13] Yildirim M Geçer E Akgül Ö. The impacts of vulnerability, perceived risk, and fear on preventive behaviours against COVID-19. Psychol Health Med. (2021) 26:35–43. 10.1080/13548506.2020.1776891 32490689

[14] Simione L Vagni M Gnagnarella C Bersani G Pajardi D. Mistrust and beliefs in conspiracy theories differently mediate the effects of psychological factors on propensity for COVID-19 vaccine. Front Psychol. (2021) 12:683684. 10.3389/fpsyg.2021.683684 34305736 PMC8292632

[15] Gallè F Sabella EA Roma P Da Molin G Diella G Montagna MT. Acceptance of COVID-19 vaccination in the elderly: a cross-sectional study in Southern Italy. Vaccines. (2021) 9:1222. 10.3390/vaccines9111222 34835152 PMC8618111

[16] Larson HJ Clarke RM Jarrett C Eckersberger E Levine Z Schulz WS. Measuring trust in vaccination: a systematic review. Hum Vaccines Immunother. (2018) 14:1599–609. 10.1080/21645515.2018.1459252 29617183 PMC6067893

[17] Falcone R Colì E Felletti S Sapienza A Castelfranchi C Paglieri F. All we need is trust: how the COVID-19 outbreak reconfigured trust in Italian public institutions. Front Psychol. (2020) 11:561747. 10.3389/fpsyg.2020.561747 33132966 PMC7562978

[18] Greyling T Rossouw S. Positive attitudes towards COVID-19 vaccines: a cross-country analysis. PLoS ONE. (2022) 17:e0264994. 10.1371/journal.pone.0264994 35271637 PMC8912241

[19] Mønsted B Lehmann S. Characterizing polarization in online vaccine discourse-A large-scale study. PLoS ONE. (2022) 17:e0263746. 10.1371/journal.pone.0263746 35139121 PMC8827439

[20] Lanyi K Green R Craig D Marshall C. COVID-19 vaccine hesitancy: analysing twitter to identify barriers to vaccination in a low uptake region of the UK. Front Digit Health. (2021) 3:804855. 10.3389/fdgth.2021.804855 35141699 PMC8818664

[21] Lentzen M-P Huebenthal V Kaiser R Kreppel M Zoeller JE Zirk M. A retrospective analysis of social media posts pertaining to COVID-19 vaccination side effects. Vaccine. (2022) 40:43–51. 10.1016/j.vaccine.2021.11.052 34857421 PMC8611612

[22] Durmaz N Hengirmen E. The dramatic increase in anti-vaccine discourses during the COVID-19 pandemic: a social network analysis of Twitter. Hum Vaccines Immunother. (2022) 18:2025008. 10.1080/21645515.2021.2025008 35113767 PMC8993086

[23] Kuckartz U. Qualitative Text Analysis: A Guide to Methods, Practice and Using Software. London: Sage (2014).

[24] Martin S Kilich E Dada S Kummervold PE Denny C Paterson P Larson HJ. “Vaccines for pregnant women…?! Absurd” – Mapping maternal vaccination discourse and stance on social media over six months. Vaccine. (2020) 38:6627–37. 10.1016/j.vaccine.2020.07.072 32788136

[25] Kuttschreuter M Gutteling JM de Hond M. Framing and tone-of-voice of disaster media coverage: the aftermath of the Enschede fireworks disaster in the Netherlands. Health Risk Soc. (2011) 13:201–20. 10.1080/13698575.2011.558620

[26] Association of Internet Researchers Ethics Working Committee. Ethical Decision-Making and Internet Research: Recommendations from the AoIR Ethics Working Committee (Version 2.0). Available online at: https://aoir.org/reports/ethics2.pdf (accessed November 26, 2021).

[27] Association of Internet Researchers Ethics Working Committee. Internet Research: Ethical Guidelines 3.0. Available online at: https://aoir.org/reports/ethics3.pdf (accessed November 26, 2021).

[28] Noelle-Neumann E. The spiral of silence a theory of public opinion. J Commun. (1974) 24:43–51. 10.1111/j.1460-2466.1974.tb00367.x

[29] McCombs ME Shaw DL. The agenda-setting function of mass media. Public Opin Q. (1972) 36:176–87. 10.1086/267990

[30] della Salute M. Vaccinazioni dell'età pediatrica e dell'adolescenza - Coperture vaccinali. Available online at: https://www.salute.gov.it/portale/documentazione/p6_2_8_3_1.jsp?lingua=italiano&id=20 (accessed October 27, 2021).

[31] Russo R Bozzola E Palma P Corsello G Villani A. Pediatric routine vaccinations in the COVID 19 lockdown period: the survey of the Italian Pediatric Society. Ital J Pediatr. (2021) 47:72. 10.1186/s13052-021-01023-6 33761977 PMC7988631

[32] WHO and UNICEF Warn of a Decline in Vaccinations During COVID-19. Available online at: https://www.who.int/news/item/15-07-2020-who-and-unicef-warn-of-a-decline-in-vaccinations-during-covid-19 (accessed October 27, 2021).

[33] Santoli JM Lindley MC DeSilva MB Kharbanda EO Daley MF Galloway L. Effects of the COVID-19 pandemic on routine pediatric vaccine ordering and administration — United States, 2020. MMWR Morb Mortal Wkly Rep. (2020) 69:591–3. 10.15585/mmwr.mm6919e2 32407298

[34] Cinelli M De Francisci Morales G Galeazzi A Quattrociocchi W Starnini M. The echo chamber effect on social media. Proc Natl Acad Sci USA. (2021) 118:e2023301118. 10.1073/pnas.2023301118 33622786 PMC7936330

[35] Schmidt AL Zollo F Scala A Betsch C Quattrociocchi W. Polarization of the vaccination debate on Facebook. Vaccine. (2018) 36:3606–12. 10.1016/j.vaccine.2018.05.040 29773322

[36] Cossard A Morales GDF Kalimeri K Mejova Y Paolotti D Starnini M. Falling into the echo chamber: the Italian vaccination debate on Twitter. In: Proceedings of the International AAAI Conference on Web and Social Media. (2020). p. 130–40.

[37] Gesualdo F D'Ambrosio A Agricola E Russo L Campagna I Ferretti B. How do Twitter users react to TV broadcasts dedicated to vaccines in Italy? Eur J Public Health. (2020) 30:481–6. 10.1093/eurpub/ckaa022 32073598 PMC7292342

[38] Tavoschi L Quattrone F D'Andrea E Ducange P Vabanesi M Marcelloni F. Twitter as a sentinel tool to monitor public opinion on vaccination: an opinion mining analysis from September 2016 to August 2017 in Italy. Hum Vaccines Immunother. (2020) 16:1062–9. 10.1080/21645515.2020.1714311 32118519 PMC7227677

[39] Ajovalasit S Dorgali VM Mazza A d'Onofrio A Manfredi P. Evidence of disorientation towards immunization on online social media after contrasting political communication on vaccines. Results from an analysis of Twitter data in Italy. PLoS ONE. (2021) 16:e0253569. 10.1371/journal.pone.0253569 34242253 PMC8270452

[40] Giambi C Fabiani M D'Ancona F Ferrara L Fiacchini D Gallo T. Parental vaccine hesitancy in Italy – results from a national survey. Vaccine. (2018) 36:779–787. 10.1016/j.vaccine.2017.12.074 29325822

[41] Fernández-Ardèvol M Belotti F Ieracitano F Mulargia S Rosales A Comunello F. “I do it my way”: idioms of practice and digital media ideologies of adolescents and older adults. New Media Soc. (2022) 24:31–49. 10.1177/1461444820959298

[42] Lovari A. Spreading (dis) trust: Covid-19 misinformation and government intervention in Italy. Media Commun. (2020) 8:458–61. 10.17645/mac.v8i2.3219

[43] Parisi L Comunello F Amico A. Networked volunteering during the 2013 Sardinian floods. Participations. J Audience Reception Stud. (2020) 17:172–96.

[44] Neiger BL Thackeray R Burton SH Thackeray CR Reese JH. Use of Twitter among local health departments: an analysis of information sharing, engagement, and action. J Med Internet Res. (2013) 15:e177. 10.2196/jmir.2775 23958635 PMC3758023

[45] Thackeray R Neiger BL Smith AK Van Wagenen SB. Adoption and use of social media among public health departments. BMC Public Health. (2012) 12:242. 10.1186/1471-2458-12-242 22449137 PMC3331826

[46] Miller MR Snook WD Yoder EW. Social media in public health departments: a vital component of community engagement. J Public Health Manage Pract. (2020) 26:94–6. 10.1097/PHH.0000000000001125 31764576

[47] National Association of County and City Health Officials (NACCHO). Social Media Toolkit A Primer for Local Health Department PIOs and Communications Professionals. Available online at: https://www.naccho.org/uploads/downloadable-resources/Social-Media-Toolkit-for-LHDs-2019.pdf (accessed November 26, 2021).

[48] Digital 2021 - I dati italiani. We Are Social Italia. (2021). Available online at: https://wearesocial.com/it/blog/2021/02/digital-2021-i-dati-italiani/ (accessed November 24, 2021).

