# Supplementary material for: How the Italian Twitter Conversation on Vaccines Changed During the First Phase of the Pandemic: A Mixed-Method Analysis
Source: Front Public Health. 2022 May 18;10:824465. doi: 10.3389/fpubh.2022.824465 (PMC9157769; doi:10.3389/fpubh.2022.824465)
Supplement: Supplementary file 1 [file Table_1.DOCX]

**Supplementary Table. Characteristics of tweets by kind of vaccine mentioned**

|  | Kind of vaccine | | | |  | |
| --- | --- | --- | --- | --- | --- | --- |
|  | Other  (n=1029) | | COVID-19  (n=1434) | | Total  (n=2463) | |
|  | n | % | n | % | n | % |
| **Month** |  |  |  |  |  |  |
| Nov 2019 | 169 | 16.4% | 0 | - | 169 | 6.7% |
| Dec 2019 | 235 | 22.8% | 0 | - | 235 | 9.5% |
| Jan 2020 | 216 | 21.0% | 21 | 1.5% | 237 | 9.6% |
| Feb 2020 | 55 | 5.3% | 110 | 7.7% | 165 | 6.7% |
| Mar 2020 | 68 | 6.6% | 334 | 23.3% | 402 | 16.3% |
| Apr 2020 | 109 | 10.6% | 502 | 35.0% | 611 | 24.8% |
| May 2020 | 88 | 8.6% | 270 | 18.8% | 358 | 14.5% |
| Jun 2020 | 89 | 8.7% | 197 | 13.7% | 286 | 11.6% |
| **Population target** |  |  |  |  |  |  |
| Children | 253 | 24.6% | 1 | 0.1% | 254 | 10.3% |
| Adult | 105 | 10.2% | 9 | 0.6% | 114 | 4.6% |
| No population target | 669 | 65.1% | 1424 | 99.3% | 2093 | 85.0% |
| **Stance** |  |  |  |  |  |  |
| Promotional | 266 | 25.9% | 533 | 37.2% | 799 | 32.4% |
| Ambiguous | 22 | 2.1% | 40 | 2.8% | 62 | 2.5% |
| Discouraging | 589 | 57.2% | 351 | 24.5% | 940 | 38.2% |
| Neutral | 106 | 10.3% | 443 | 30.9% | 549 | 22.3% |
| Other | 46 | 4.5% | 67 | 4.7% | 113 | 4.6% |
| **Tone of voice** |  |  |  |  |  |  |
| Ironic | 75 | 7.3% | 112 | 7.8% | 187 | 7.6% |
| Polemical/complaining | 619 | 60.2% | 522 | 36.6% | 1141 | 46.5% |
| Worried | 65 | 6.3% | 151 | 10.6% | 216 | 8.8% |
| Neutral | 145 | 14.1% | 441 | 30.9% | 586 | 23.9% |
| Other | 124 | 12.1% | 201 | 14.1% | 325 | 13.2% |
| **Kind of author** |  |  |  |  |  |  |
| Lay users | 844 | 87.7% | 1010 | 76.9% | 1854 | 81.5% |
| Media companies or single journalist | 50 | 5.2% | 223 | 17.0% | 273 | 12.0% |
| Other | 68 | 7.1% | 81 | 6.2% | 149 | 6.5% |
| **Information source** |  |  |  |  |  |  |
| Content published by mainstream media | 197 | 19.4% | 355 | 24.9% | 552 | 22.6% |
| Social media posts | 164 | 16.1% | 30 | 2.1% | 194 | 7.9% |
| Other | 203 | 20.0% | 143 | 10.0% | 346 | 14.2% |
| No source | 453 | 44.5% | 896 | 62.9% | 1349 | 55.3% |
